# Molecular Analysis of the Bacterial Communities in Crude Oil Samples from Two Brazilian Offshore Petroleum Platforms

**DOI:** 10.1155/2012/156537

**Published:** 2012-01-29

**Authors:** Elisa Korenblum, Diogo Bastos Souza, Monica Penna, Lucy Seldin

**Affiliations:** ^1^Laboratório de Genética Microbiana, Instituto de Microbiologia Prof. Paulo de Góes, Universidade Federal do Rio de Janeiro, Centro de Ciências da Saúde, Bloco I, Ilha do Fundão, 21941-590 Rio de Janeiro, RJ, Brazil; ^2^Gerência de Biotecnologia e Tratamentos Ambientais, CENPES-PETROBRAS, Ilha do Fundão, 21949-900 Rio de Janeiro, RJ, Brazil

## Abstract

Crude oil samples with high- and low-water content from two offshore platforms (PA and PB) in Campos Basin, Brazil, were assessed for bacterial communities by 16S rRNA gene-based clone libraries. RDP Classifier was used to analyze a total of 156 clones within four libraries obtained from two platforms. The clone sequences were mainly affiliated with *Gammaproteobacteria* (78.2% of the total clones); however, clones associated with *Betaproteobacteria* (10.9%), *Alphaproteobacteria* (9%), and Firmicutes (1.9%) were also identified. *Pseudomonadaceae* was the most common family affiliated with these clone sequences. The sequences were further analyzed by MOTHUR, yielding 81 operational taxonomic units (OTUs) grouped at 97% stringency. Richness estimators also calculated by MOTHUR indicated that oil samples with high-water content were the most diverse. Comparison of bacterial communities present in these four samples using LIBSHUFF and Principal Component Analysis (PCA) indicated that the water content significantly influenced the community structure only of crude oil obtained from PA. Differences between PA and PB libraries were observed, suggesting the importance of the oil field as a driver of community composition in this habitat.

## 1. Introduction

Biodegraded oils resulting from the action of microorganisms that destroy hydrocarbons and other oil components have been a problem for the petroleum industry. Biodegradation is responsible for the increase in viscosity and acidity of the oil and the reduction of its API (American Petroleum Institute) grade [1]. Different studies have already demonstrated the existence of large and diverse populations of microbes with different metabolic activities in petroleum systems [2–5], but due to difficulties in sampling and in the efficiency of DNA extraction from oily samples, little is known about the bacterial diversity in crude oil [6–8]. It is also challenging to work with samples with low indigenous biomass as well as a high, oily, and viscous emulsion.

 During the aging of oil fields, industries make use of secondary oil recovery (SOR), which consists of water injection inside the reservoir to maintain the formation pressure, resulting in an increase of water content in crude oil (oil : water ratio of the production fluids). SOR and high-water content in the petroleum reservoir may reduce internal temperature and allow the biodegradation of crude oil by autochtone or allochtone bacterial populations [9]. To control the bacterial contamination in the reservoir and subsequently in the production line, petroleum industries use physical and chemical treatments of water injected into the reservoir during SOR [10]. In Brazil, seawater is used in SOR at offshore platforms. The seawater is treated by filtration, chlorination, deoxygenation, and/or biocide addition to reduce bacterial contamination prior to water injection. However, these treatments do not completely eliminate the bacterial contamination from the injection water [8]. Besides waterflooding during SOR, other sources of contamination to the system are drilling operation, well equipment, and damaged tubing or casings. Hence, exogenous bacteria can penetrate the reservoir, form biofilms inside the rock and in the oil-producing line and be recovered at producing wells [2, 11]. Therefore, microbiology in crude oil samples might reflect the indigenous organisms in the respective oil formations and also, to a significant extent, allochthonous bacterial populations.

 Despite the recent use of molecular techniques for a broader survey of microbial communities in oil fields, our knowledge of the nature and diversity of bacteria present in these ecosystems is still scarce, especially in waterflooded oil reservoirs. Therefore, in this study, the bacterial communities from crude oil samples containing high- and low-water content from two platforms in Campos Basin were analyzed using the 16S rRNA gene sequencing approach in order to determine whether the different water content could influence the bacterial communities from crude oils and, consequently, pose a risk of decreasing oil quality.

## 2. Materials and Methods

### 2.1. Samples

The Caratinga and Barracuda oil fields are located in the south-central portion of the Campos Basin approximately 90 km offshore of Rio de Janeiro State, Brazil, at water depths ranging between 600 and 1200 m. The Caratinga and Barracuda oil fields are denoted as platform A (PA) and platform B (PB), respectively. These two oil fields cover a total area of approximately 234 km^2^. The oil densities from these fields range from 20 to 26 API degrees. The temperatures of the Caratinga and Barracuda fields at 2,800 m below sea level are 78 and 79.5°C, respectively. Seawater was injected into these platforms in 2005 for SOR. Two wellheads were selected within each platform, one with high-water content and another with low-water content. The wellheads were denoted as PAH, PAL, PBH, or PBL, corresponding to platform PA or PB and high- (H) or low- (L) water content (PAH, 60% water content; PAL, 5%; PBH, 40%; and PBL, 1%). The crude oil samples were collected in sterile 1 L flasks directly from each platform wellhead after enough crude oil was drained off to clean the wellheads. Samples were stored at 4°C until their arrival at the laboratory.

### 2.2. DNA Extraction

Crude oil samples (10 mL) were mixed with an equal volume of Winogradsky buffer [7] and incubated for 10 min at 80°C. The aqueous phase was recovered with a sterile pipette. This procedure was repeated five times, collecting a total volume of 50 mL of aqueous phase for each sample. The total volume of aqueous phase for each sample was pelleted by centrifugation at 12,000 ×g for 20 min at 4°C. An aliquot (2 mL) of TE buffer (10 mM Tris; 1 mM EDTA) was added to the pellet, and DNA extraction was further performed as described previously [12]. In order to exclude the possibility of bacterial contamination from the reagents, buffers and/or enzymes used, DNA extraction of a blank tube without sample was additionally carried out.

### 2.3. Polymerase Chain Reaction Conditions for Bacterial 16S rRNA Gene Amplification

16S rRNA gene sequences were amplified by polymerase chain reaction (PCR) using the primers and the PCR conditions described previously [13]. Primers U968 and L1401 were used to amplify the V6–V8 variable regions in the *Escherichia coli* small subunit rRNA genes. The 50 *μ*L PCR reaction contained 50 mM KCl, 2.5 mM MgCl_2_, 2 mM dNTPs, 0.2 *μ*M of each primer (U968: 5′AACGCGAAGAACCTTAC3′ and L1401: 5′GCGTGTGTACAAGACCC3′), 2.5 U of *Taq* DNA polymerase (Promega, Madison, WI), and 1 *μ*L (20–50 ng) of the DNA extract. The amplification conditions included a denaturing step at 94°C for 2 min followed by 35 cycles at 94°C for 1 min, an annealing step at 48°C for 1 min and 30 s, and an extension step at 72°C for 1 min and 30 s; the final extension step was performed at 72°C for 10 min. Negative controls (without DNA) were also included in all sets of PCR reactions. PCR products were then purified using the Wizard PCR Clean-Up System (Promega) and eluted with 50 *μ*L of distilled water. The presence of PCR products was confirmed by 1.4% agarose gel electrophoresis.

### 2.4. Molecular Analyses

The partial 433-bp 16S rRNA gene sequences obtained by PCR were cloned using the pGEM T-easy vector according to instructions from the manufacturer (Promega). The resulting ligation mixtures were transformed into *Escherichia coli* JM109 competent cells, and clones containing inserts were sequenced. All sequencing reactions were performed by Macrogen Inc. (South Korea). The sequence data set was screened for potential chimeric structures by using Bellerophon [14] (available at the greengenes website (version 3, http://greengenes.lbl.gov/cgi-bin/nph-bel3_interface.cgi)). A total of 156 valid 16S rRNA gene sequences were analyzed for taxonomic affiliation by the RDP Classifier [15] and for the closest match to sequences in the GenBank database by BLASTN [16]. The 16S rRNA gene sequences were submitted to GenBank with the accession numbers: HQ341990–HQ3411999, HQ342010, HQ342021, HQ342032, HQ342043, HQ342054, HQ342064–HQ342083, HQ342084–HQ342121, HQ342122–HQ342146, HQ342000–HQ342009, HQ342011–HQ342017, HQ342018–HQ342031, HQ342033–HQ342039, HQ342041–HQ342053, and HQ342055–HQ342063.

 16S rRNA gene sequences were then clustered as OTUs at an overlap identity cut-off of 97% by MOTHUR software [17]. Richness and diversity statistics including the nonparametric richness estimators ACE, Chao1, and Shannon diversity index were calculated also using MOTHUR. The diversity of OTUs and community overlap were examined using rarefaction analysis and Venn diagram. A phylogenetic tree was constructed with representatives of each OTU found within the four libraries (at a distance level of 3%) and with closely related sequences that were recovered from the GenBank database. Sequence alignment was done by Clustal-X software [18], and the aligned sequences were then used to construct the phylogenetic tree with the neighbor-joining method by using the MEGA5 software [19]. Bootstrap analyses were performed with 1,000 repetitions, and only values higher than 50% are shown in the phylogenetic tree.

### 2.5. Statistical Analyses

Based on the sequence alignment mentioned above, a distance matrix was constructed using DNAdist from PHYLIP (version 3.6) [20], and pairwise comparisons of each clone library were performed using LIBSHUFF (version 0.96; http://www.mothur.org/wiki/Libshuff) [21]. Additionally, the relationship among bacterial community structures was evaluated using the Principal Component Analysis (PCA). The matrix used for the PCA was a quantitative matrix of abundance of all OTUs detected from each clone library.

## 3. Results

### 3.1. 16S rRNA Gene Sequence Analysis

**Figure 1:**
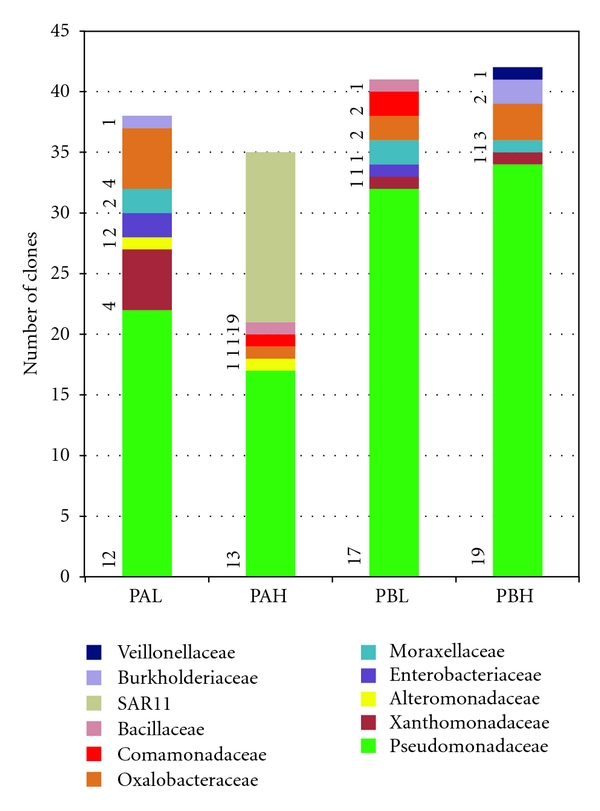
Frequency of clones affiliated with different bacterial families found in each oil sample from platforms A and B with low- (L) and high- (H) water contents. A total of 156 16S rRNA gene clones were classified by the RDP Classifier tool. The number of OTUs corresponding to each family is indicated close to the graph bar. OTUs were defined using a distance level of 3% by using the furthest neighbor algorithm in MOTHUR. PAL—crude oil from Platform A with low-water content (5%); PAH—crude oil from Platform A with high-water content (60%); PBL—crude oil from Platform B with low water content (1%); PBH—crude oil from Platform B with high water content (40%).

After recovering the microbial fraction, DNA was successfully extracted from the crude oil samples. However, a low amplicon yield was obtained with the bacterial 16S rRNA gene primers used, probably due to the inefficiency of DNA extraction from oily samples. As expected, no amplicons were obtained from the blank tube. The PCR products were cloned, and a total of 156 valid 16S rRNA gene sequences were analyzed for taxonomic affiliation by the RDP Classifier.  Figure 1 shows the bacterial clone frequencies obtained in the PAH (35 clones), PAL (38 clones), PBH (42 clones), and PBL (41 clones) samples and the clone affiliation. The number of OTUs corresponding to each family is indicated close to the bars on the graph (Figure 1).

Among the clones, 122 (78.2%) were *Gammaproteobacteria,* and most were associated with the family *Pseudomonadaceae* (105 clones). The remaining clones (17) were affiliated with *Moraxellaceae*, *Enterobacteriaceae*, *Alteromonadaceae,* and *Xanthomonadaceae*. Also, 17 (10.9%) of the total clones were *Betaproteobacteria,* and most were associated with the families *Comamonadaceae* (3 clones from samples PAH and PBL), *Burkholderiaceae* (3 clones from PAL and PBH), and *Oxalobacteraceae* (11 clones from samples PAH, PAL, PBH, and PBL). A few clones (14) from the PBH sample, corresponding to 9% of the total amount of clones analyzed, were affiliated with SAR11 clade, a lineage of bacteria from the *Alphaproteobacteria* class, which is common in the ocean [22]. To a lesser extent, clones from *Bacillaceae *(two clones, one of each sample PAH and PBL) and* Veillonellaceae *(one clone from sample PBH) were present, representing the Firmicutes.

**Figure 2:**
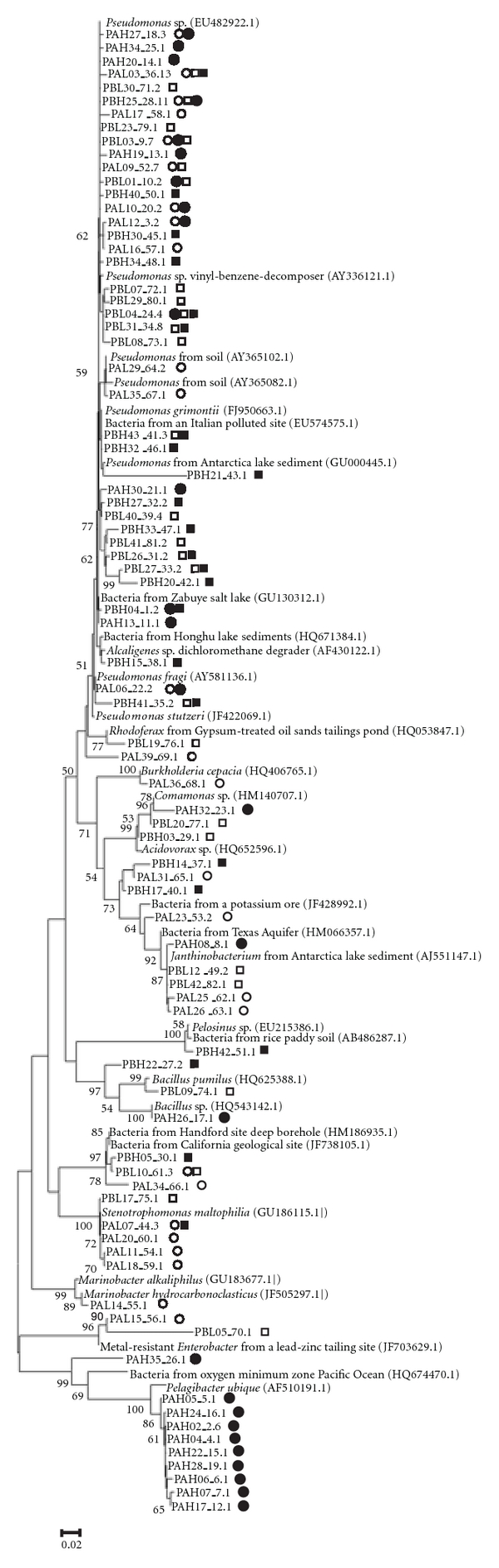
Phylogenetic tree of the 16S rRNA gene-based libraries obtained from platforms A and B. OTUs were defined by MOTHUR using the furthest neighbor algorithm with a distance level of 3%. One representative clone of each OTU (81 OTUs) was used for phylogenetic analysis. Reference sequences from GenBank are highlighted in bold. The tree was constructed based on the neighbor-joining method. Bootstrap analyses were performed with 1,000 repetitions and only values higher than 50% are shown. The scale bar indicates the distance in substitutions per nucleotide. Clones indicated with prefixes PAH and PAL originated from platform A with high- and low-water content, respectively. Clones PBH and PBL were obtained from platform B with high- and low-water content, respectively. The access name of each sequence is formed by numbers that correspond to the following: a representative clone number, followed by the OTU and the number of clones in that OTU (e.g., sequence PAH27_18, 3 = name of the clone_OTU, number of clones grouped in this OTU). The symbols after each access name indicate that the OTU was found in oil samples from PAH (*⚫*), PAL (○), PBH (■), and/or PBL (□).

All 16S rRNA gene sequences were then clustered as OTUs, and using the 81 resulting OTUs, a phylogenetic tree was constructed with the closely related sequences that were recovered from the GenBank database (Figure 2). The phylogenetic tree showed that the majority of OTUs from Platforms A and B fall within *Gammaproteobacteria*.

### 3.2. Diversity Analyses

**Figure 3:**
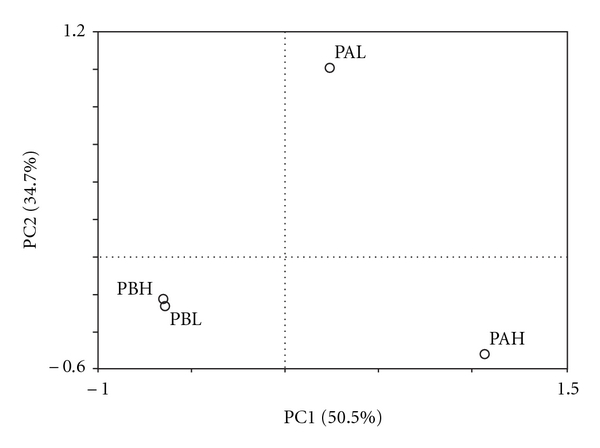
Comparison of bacterial communities in samples PAH, PAL, PBH, and PBL. Principal coordinates plots (PCA) were generated using the presence of each OTU (at a distance level of 3%) found in each clone library. PAL—crude oil from Platform A with low-water content (5%); PAH—crude oil from Platform A with high-water content (60%); PBL—crude oil from Platform B with low-water content (1%); PBH—crude oil from Platform B with high-water content (40%).

Sequences obtained from the different sampling sites were evaluated by pairwise analysis with LIBSHUFF. Crude oil samples with high water content values (PAH and PBH) were statistically different (*P* = 0.001). PAL and PAH libraries also were statistically different. However, LIBSHUFF did not show statistical differences between PB libraries (PBL and PBH) nor between samples with low water content (PAL and PBL). This latter finding was similar to the PCA result (Figure 3). The PCA grouped together PBH and PBL samples, whereas the PAH and PAL samples diverged in this analysis. In addition, the first component determined 50.5% of the total variation, showing a dichotomic separation of the crude oil sample from PAH and the three other samples (PAL, PBL and PBH).

**Figure 4:**
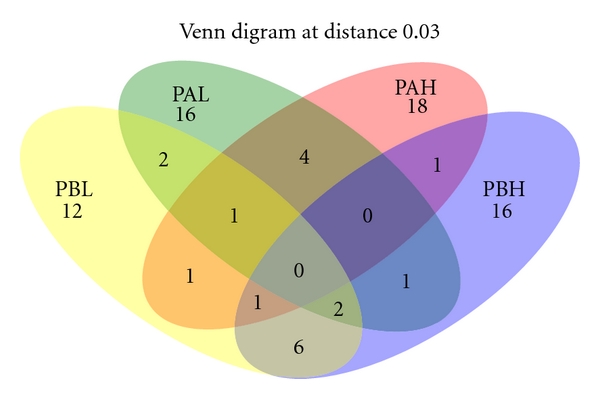
Venn diagram of bacterial OTUs clustered with a 3% distance threshold, showing the number of OTUs shared by the four crude oil samples. PAL—crude oil from Platform A with low-water content (5%); PAH—crude oil from Platform A with high-water content (60%); PBL—crude oil from Platform B with low-water content (1%); PBH—crude oil from Platform B with high-water content (40%).

 The number of OTUs from each sampling site as well as richness and diversity indexes are shown in Table 1. Total coverage of bacterial richness was almost achieved in all libraries (data not shown). Libraries from oil samples with high water content (PAH and PBH) had a higher richness based on ACE and Chao1 than PAL and PBL. While the Shannon diversity index showed that bacterial communities are 97% similar, the Venn diagram showed that no OTUs are shared between all four samples, indicating that the bacterial communities are different in these two platforms (Figure 4).

## 4. Discussion

Barracuda and Caratinga are giant oil fields from the deep water Campos Basin, which is one of the most economically important petroleum basins in Brazil. The bacterial communities in crude oil samples from these two platforms were assessed for the first time in this study. Moreover, the difference in the water content of the oil samples was considered in each platform. Water content may influence the bacterial diversity and biodegradation of oil in the reservoir. The latter process requires not only water but also nutrients and hydrocarbons for microbial growth [4]. Biodegraded oil reservoirs usually produce oil-water emulsified fluids, which may complicate the recovery of the totality of the microorganisms present in those samples. Oil from the Caratinga and Barracuda fields ranges from 20 to 26 API degrees, values corresponding to low/medium petroleum degradation. The results obtained in this study indicate that these fields may have a potential for biodegradation because of the bacteria found in the oil samples.

 An alternative to microorganism isolation and cell counts, one of the main strategies to study the microbial diversity, is the PCR-based approach, which also may provide an understanding of the uncultured microbial community. However, crude oil samples contain low amounts of biomass that may result in a low DNA yield, which affects the efficiency of the molecular methods [23]. These PCR-based techniques are also highly prone to DNA contamination and can result in false positive amplifications. To overcome these problems in this study, the oil samples were washed several times with Winogradsky buffer to recover bacterial cells and to obtain higher amounts of biomass prior to DNA extraction [7]. Low but sufficient amounts of bacterial DNA for PCR amplification in these oil samples were achieved. Negative controls were also included during the DNA extraction and PCR amplification procedures, and no amplicons were obtained in these controls.

Data obtained in this study show a spatial heterogeneity of bacterial community composition in Campos Basin, comparing Caratinga (PA) and Barracuda (PB) fields. In addition, water injection might have stimulated bacterial growth by supplying nutrients and decreasing downhole temperature, as high-water content samples (PAH, and PBH) showed higher richness index values (Table 1). Most libraries were not statistically different from each other, except for PAH. The difference in the amount of water in PBL (1% water content) and PBH (40% water content) seemed not to be large enough to significantly affect the community structure. When PAH (which had the highest water content—60%) was compared with PAL (5% water content), as well as when PAH was matched up to PBH, a shift of bacterial diversity was observed. These data were also observed by PCA, where PC1 represents the OTUs spatial distribution, separating PA and PB, while PC2 represents the water content, which influenced only PAL and PAH. PAH divergence may be due to an abundant group that was observed only in this library. Almost half of PAH clones were related to *Pelagibacter*. This genus belongs to the SAR11 clade, which is a very small, heterotrophic marine *Alphaproteobacteria* found throughout the oceans [22]. Therefore, we believe that this bacterium may have been introduced with the seawater during secondary oil recovery, not being indigenous of the reservoir.

An abundant group belonging to the family *Pseudomonadaceae* was observed in the four 16S rRNA gene libraries. The presence of this group may be a potential risk for petroleum biodegradation inside the reservoir, because members of this family have been isolated and also detected by molecular techniques in deep ocean crust and in high temperature oil reservoirs [24, 25]. Moreover, the genus *Pseudomonas* has been shown to degrade alkanes and other polyaromatic hydrocarbons as well as to emulsify and degrade resins from Arabian light crude oil [26]. Nelson et al. [27] demonstrated that the species *P. putida* is a metabolically versatile bacterium that is able to degrade a wide variety of xenobiotic compounds. Also, Chayabutra and Ju [28] showed that another member of this genus, *P. aeruginosa*, degrades *n*-hexadecane under anaerobic denitrifying conditions.

In addition to *Pseudomonadaceae*, clone sequences affiliated with *Moraxellaceae*, *Enterobacteriaceae*, *Alteromonadaceae,* and *Xanthomonadaceae* families were also found in the oil samples studied. Some members of these families have been shown to be involved in hydrocarbon degradation [29–32]. *Betaproteobacteria*-related clones belonging to the *Burkholderiales* order were observed in crude oil samples from Barracuda and Caratinga fields. Some genera of this group (*Burkholderia* and *Comamonas*) have been previously found to grow under anaerobic conditions in petroleum reservoirs or in contaminated soils [1, 33]. Therefore, the presence of these groups in the oil reservoirs studied may suggest marine bacterial contamination after the water injection used for SOR. Korenblum et al. [8] have also detected *Marinobacter*, *Burkholderia,* and *Pseudomonas* in water used in SOR.

Few clones related to the Firmicutes phylum were detected in samples PAH, PBH, and PBL. Within this phylum, *Bacillus *strains have been previously isolated from oil reservoirs in Brazil [8, 34], where different strains have been shown to degrade petroleum hydrocarbons [34]. However, this is the first time the presence of another genus of Firmicutes, a *Pelosinus*-related clone, was observed in a petroleum environment. *Bacillus* and *Pelosinus* species may also survive in a petroleum environment due to spore formation or use of nitrate or iron for respiration.

In conclusion, we report the bacterial composition present in crude oil samples with high- and low-water content from two Brazilian oil fields, Caratinga and Barracuda. This information on bacterial diversity in crude oil increases the current knowledge of the microbial ecology in this environment, which may help to predict the potential for biodegradation in these fields. Based on their 16S rRNA sequences, most of the clones obtained were related to different bacterial families, with a predominance of *Pseudomonadaceae*, which was observed in all crude oil samples. Moreover, few clones were related to genera that have not been described before in this environment. However, we are aware that a limited number of clones were evaluated in this study. Bacterial isolation is still necessary to determine the industrial and ecological significance of these different bacteria.

## Figures and Tables

**Table 1:** Species richness estimates and diversity of 16S rRNA gene clones calculated by MOTHUR.

	Samples^a^			
	PAL	PAH	PBL	PBH
OTUs^b^	26	26	25	27
ACE^c^	50	122	40	306
Chao1	34	100	38	88
H^'d^	2.94	2.94	2.88	2.87

^a^PAL—crude oil from Platform A with low water content (5%); PAH—crude oil from Platform A with high-water content (60%); PBL—crude oil from Platform B with low-water content (1%); PBH—crude oil from Platform B with high-water content.

^b^Number of unique OTUs defined by using the furthest neighbor algorithm by MOTHUR at 97% stringency.

^c^ACE (Abundance-based coverage estimator).

^d^H^'^ (Shannon-weaver index of diversity).
